# Why do we use 100 mg of clofazimine in TB and NTM treatment?

**DOI:** 10.1093/jac/dkae041

**Published:** 2024-02-22

**Authors:** Jakko van Ingen

**Affiliations:** Department of Medical Microbiology (777), Radboudumc Community for Infectious Diseases, Radboud University Medical Center, PO Box 9101, 6500 HB, Nijmegen, The Netherlands

## Abstract

Current tuberculosis and non-tuberculous mycobacterial disease guidelines recommend the use of clofazimine in a 100 mg once-daily dose. The rationale behind this exact dose is not provided.

I performed a literature review to determine the reasoning behind the current dosing regimen. The current 100 mg once-daily dose of clofazimine stems from a deliberate attempt to find the minimum effective daily dose in leprosy treatment, driven by efficacy, economical and toxicity considerations.

While this dose is safe, economical and practical, a higher dose with a loading phase may add relevant efficacy and treatment-shortening potential to both tuberculosis and non-tuberculous mycobacterial disease treatment. We need to revisit dose–response and maximum tolerated dose studies to get the best out of this drug, while continuing efforts to generate more active r-iminophenazine molecules that accumulate less in skin and intestinal tissues and have pharmacokinetic properties that do not require loading doses.

## Introduction

Clofazimine, or G30,320 or B663 as it was first known, is an r-iminophenazine class molecule that was discovered in 1957 and was first used in leprosy clinical trials in 1962.^1,2^ Today it is an integral part of multidrug therapy (MDT) for multibacillary leprosy as well as an important (group B) drug in MDR-TB treatment^3^ and an increasingly important drug in treatment regimens for non-tuberculous mycobacterial (NTM) disease.^4,5^

In leprosy, the standard dose in the treatment of multibacillary leprosy is 300 mg once a month, and 50 mg once daily.^6^ In TB and NTM treatment, the standard dose is 100 mg once daily for adults,^3–5^ but the peculiar pharmacokinetics of the extremely lipophilic clofazimine determine that, with the 100 mg once-daily dose, the steady state is only reached after 4 months of treatment.^7^ Hence, recent modelling studies have suggested that a 200 or 300 mg loading dose for the first 6–8 weeks may be used to shorten the time to steady state and thus the time to efficacy of clofazimine.^7^ Achieving adequate exposures fast seems important, as clofazimine shows exposure-driven sterilizing and a treatment-shortening effect in TB in mouse models^8,9^ and in patients with MDR-TB.^10^

The rationale behind the 100 mg once-daily dose is not provided in any of the current guidelines.^4,5,11^ Hence, I performed a literature review focused on the development stage and early clinical application phase of clofazimine, to determine the reasoning behind the current dosing regimen.

## Methods

A PubMed (NCBI: https://pubmed.ncbi.nlm.nih.gov/) search was performed on 1 March 2023, using the following search terms: ((((((‘clofazimine/administration and dosage”[MeSH Terms]) OR (‘clofazimine/isolation and purification”[MeSH Terms])) OR (‘clofazimine/organization and administration”[MeSH Terms])) OR (‘clofazimine/therapeutic use”[MeSH Terms])) OR (‘clofazimine/pharmacokinetics”[MeSH Terms])) OR (B663 [tiab])) OR (G30320 [tiab])) NOT (‘review”[Filter]); I performed a similar search using Medline, with the search term ‘Clofazimine/ad, pk, sd’.

Search results were screened for eligibility on the basis of title and then abstract, which had to address clofazimine and dosing. Only English or Dutch language articles available as full text via the Radboud University Medical Center library or via the Interlibrary Loan service were considered for this review.

In addition, I reviewed WHO guidelines on MDR-TB and leprosy treatment, available through the WHO website (http://www.who.int).

## Results

The search yielded 834 articles, of which 63 were included after title and abstract review and 59 were available as full text. Eleven WHO guidelines or statements were considered relevant to the research question (5 leprosy documents, 6 TB documents). Dose recommendations over time are presented in Table 1.

**Table 1.** Clofazimine dosing in relevant clinical trials and guidelines

Year (First author)	Indication	Reported dosing
1965 (Browne)^2,12^	Leprosy curative treatment	300 mg/day 6×/week, 6 months
1967 (Pettit)^13^	Leprosy curative treatment	300 mg/day 6×/week, 5 months
1969 (Waters)^14^	Leprosy curative treatment	100 mg twice weekly to 200 mg od (±300 mg/day loading in first 3 weeks to 3 months)
1970 (Karat)^15^	Suppress leprosy reactions	300 mg/day, 12 weeks
1972 (Levy)^16^	Leprosy curative treatment	100–200 mg od
1973 (Revill)^17^	Buruli ulcer disease	20–30 mg/kg (800 mg od maximum)
1977 (THELEP)^18^	Leprosy curative treatment	100 mg od
1979 (THELEP)^18^	Leprosy curative treatment	600 mg daily on two consecutive days once a month
1982 WHO study group^19^	Leprosy curative treatment	300 mg once monthly, supervised, and 50 mg daily, self-administered
1997 ATS NTM guideline^20^	NTM treatment	Do not use; some use 100 mg od
2007 ATS/IDSA NTM guideline^21^	NTM treatment	Do not use
2008 WHO MDR-TB guideline^22^	MDR-TB curative treatment	100–300 mg od, consider 4–6 weeks 300 mg then 100 mg od
2010 MDR-TB Bangladesh regimen^23^	MDR-TB curative treatment	100 mg od
2016 WHO MDR-TB guideline^24^	MDR-TB curative treatment	No guidance on dosing
2018 WHO Technical Report^25^	PK/PD and dosing advice	100 mg od
2018 WHO Leprosy guideline^6^	Leprosy curative treatment	300 mg once monthly, supervised, and 50 mg daily, self-administered
2019 WHO MDR-TB guideline^3^	MDR-TB curative treatment	100 mg od
2020 NTM guideline^4^	NTM treatment	100–200 mg od
2022 NTM rare species guideline^5^	NTM treatment	100–200 mg od
2022 WHO MDR-TB guideline^11^	MDR-TB curative treatment	No guidance on dosing

THELEP, WHO working group on Leprosy Treatment; ATS, American Thoracic Society; od, once daily.

In the original description of clofazimine, Barry *et al.*^1^ noticed that a 2 mg/kg daily dose of clofazimine exerted anti-TB activity in mice, without crystal formation in their organs; a 5 mg/kg dose showed a more potent anti-TB effect, but doses over 5 mg/kg did not show additional efficacy. A 5 mg/kg daily dose for 38 days before infection with *Mycobacterium tuberculosis* was able to prevent disease.^1^ The authors provide no hypotheses on clinically relevant doses.

### Clofazimine dosing in leprosy

In 1962, Browne and Hogerzeil^2^ reported the preliminary outcomes of the first ever clinical trial of clofazimine treatment in 16 patients with leprosy, showing clinical improvement and reduced bacterial loads in all patients, especially those receiving clofazimine and dapsone. In a long-term follow-up paper they reported on all 28 patients treated for lepromatous or borderline leprosy. They dosed based on weight, with a mean of 5 mg/kg, i.e. 300 mg, once daily for 6 or 12 months.^2,12^ Soon thereafter, Pettit *et al.*^13^ reported on a similar study in which six patients with lepromatous or borderline leprosy were administered a 300 mg once-daily dose of clofazimine, 6 days per week, for 5 months. Clinical and bacteriological improvement were recorded for all patients, but the authors suggested trying lower doses as cooperation with patients had become ‘less than wholehearted’. Patients feared they would be treated with clofazimine and ‘turn an extremely unpleasant colour’.

On 26 September 1968, a WHO Working Party was held in London to exchange findings on the therapeutic use of clofazimine; the various investigators reported successful use of clofazimine in doses ranging from 200 mg per week to 200 mg per day, including use of 3 week- to 3 month-long 300 mg/day loading doses.^14^ It was concluded that 300 mg/week should suffice in mild disease, but 100 mg per day is required to treat active dapsone-resistant leprosy. Only for leprosy reactions, doses up to 600 mg per day may be required for control, followed by a tapering phase. In a 1970 opinion paper by the experts in the WHO Working Party, this quest to lower clofazimine doses without reducing its efficacy was phrased as finding the ‘minimal useful dose’.^26^

This call for lower doses was answered in clinical studies. A retrospective analysis of 11 patients with dapsone-resistant leprosy demonstrated efficacy of 200 mg once-daily doses, despite frequent dose reductions to 100 mg because of hyperpigmentation.^16^ The first trial of deliberate low dosing studied the efficacy of 100 mg of clofazimine given twice weekly.^27^ It showed that the bacteriological efficacy of low-dose clofazimine was equal to that of dapsone, but the number of reactions was lower and the skin discoloration commonly seen at higher clofazimine doses remained absent. While favouring the use of lower doses of clofazimine, the authors did echo that low-dose monotherapy may induce resistance.^27^

In 1975, the US Leprosy Panel reported on a dose-fractionation clinical study of 200 mg daily 6 days per week (1200 mg per week) versus four arms of 300 mg per week doses fractionated as 100 mg three times weekly, 300 mg once weekly, 600 mg every other week and 600 mg on two consecutive days every 4 weeks; efficacy was monitored by inoculation of skin biopsy samples in mouse footpads. Strongest and fastest antimycobacterial activity was seen in the 200 mg six times per week and the 100 mg three-times-weekly dosing regimen and worsened with longer intervals. Skin discoloration occurred in all patients, but was most marked in patients taking 1200 mg/week doses.^28^

In 1976, leprosy treatment entered a new phase with the start of the Chemotherapy for Leprosy (THELEP) Scientific Working Group at the WHO. THELEP designed and performed a series of clinical trials of multidrug treatment of leprosy. The trials tested combination regimens of 100 mg once-daily doses of clofazimine (without rationale) with rifampicin and dapsone.^18^

The final step in the evolution of clofazimine dosing in leprosy occurred in 1982 when the WHO technical report on leprosy chemotherapy stated that the best efficacy of clofazimine is obtained with daily or thrice-weekly dosing, yet monthly dosing is possible and allows for supervised intake. So, while acknowledging that a 50–100 mg once-daily dose would be efficacious, the WHO Study Group on leprosy decided that to increase its killing effect on rifampicin-resistant *Mycobacterium leprae* mutants, a supervised monthly dose of 300 mg of clofazimine was to be supplemented by daily unsupervised 50 mg doses. Although the drug cost was high, the risks of unsupervised use were deemed low.^19^ Also, the report stressed the need for additional studies on the efficacy of smaller monthly doses and to establish ‘the minimum effective daily dose’.^19^ The rationale for 300 mg once monthly plus 50 mg once daily was later explained as ‘Clofazimine is a repository drug, i.e. it is stored in the body after administration and is then slowly excreted. It is given as a loading dose of 300 mg once a month to ensure that the optimal amount of clofazimine is maintained in the body tissue, even if the patient occasionally misses his or her daily dose’.^29^

### Uptake in TB guidelines

Clofazimine first made it into the MDR-TB guidelines in 2008 as a Class 5 drug, i.e. only to be used if more effective or more evidence-based options were not possible. The guideline provides dosing recommendations, albeit imprecise as ‘100–300 mg once daily, some use 300 mg for 4–6 weeks and then continue with 100 mg’.^22^ Clofazimine’s role was reconfirmed by inclusion in the so-called ‘Bangladesh’ regimen, at a 100 mg daily dose (or 50 mg for those <33 kg body weight).^23^

In the 2016 WHO guideline, clofazimine moved up to group C status and represented a core second-line MDR-TB drug.^24^ In 2018, a WHO working group on pharmacokinetics/pharmacodynamics of anti-TB drugs sought to formulate dosing advice for clofazimine but concluded that ‘available data preclude a conclusive position regarding the optimal dose and dosing frequency of clofazimine for shorter and longer MDR-TB regimens. Most of the experience in its use has been from observational studies of the shorter MDR regimens, in which most patients received 100 mg/day. Given the absence of other evidence showing improved benefit-to-harm in MDR-TB patients, 100 mg/day (50 mg/day for individuals <40 kg body weight), without a loading dose, is the recommended dose’.^25^ Early experiences in leprosy treatment were not considered.

In 2018, an individual patient data metanalysis project demonstrated that, among 12 030 patients of whom 834 used clofazimine, MDR-TB treatment success was positively associated with the use of clofazimine.^30^ As a result, in the 2019 WHO guidelines, clofazimine moved up to group B, i.e. most individualized MDR-TB regimens should include clofazimine and the recommended dose was 100 mg once daily for patients weighing >30 kg.^3^ In the 2022 update, no dosing recommendations are given.^11^

### Clofazimine dosing in NTM disease

Clofazimine was tried without success in a clinical trial in disseminated *Mycobacterium avium* complex (MAC) disease in 106 people living with HIV/AIDS (100 mg once daily, no rationale for dose given) in the 1990s.^20,31^ The guideline on NTM disease treatment published in 1997 recommends not to use clofazimine for disseminated MAC disease, but state that ‘some experts feel that clofazimine 100 mg/day is useful in the context of *M. avium* complex lung diseases, although there are no data corroborating its efficacy’.^20^ Those data came soon after, when two clinical cohort studies demonstrated that a clofazimine/minocycline/clarithromycin regimen (in 30 patients)^32^ and a clofazimine/ethambutol/clarithromycin regimen (also in 30 patients)^33^ yielded outcomes comparable to recommended rifamycin/ethambutol/macrolide regimens. In both cohorts, clofazimine was dosed at 100 mg once daily, without explanation. These observations were insufficient to recommend clofazimine use in MAC pulmonary disease (MAC-PD) in the 2007 ATS/IDSA guidelines, which only reiterated that clofazimine should not be used in disseminated MAC disease and that its role in MAC-PD treatment was not established.^21^

The view on the potential of clofazimine changed when a larger-scale (93 patients on clofazimine-containing regimens) follow-up study in Canada showed that culture conversion rates of the clofazimine/ethambutol/macrolide regimens were in line with, or even better than, those of recommended rifampicin-containing regimens.^34^ Simultaneously, retrospective studies showed that adding clofazimine and amikacin to rifampicin/ethambutol/macrolide regimens for severe MAC-PD yielded good outcomes^35^ and that incorporating clofazimine in refractory *Mycobacterium abscessus* disease treatment showed positive signals.^36^ All these clinical cohort studies used the 100 mg once-daily dose. *In vitro* studies added that clofazimine can prevent macrolide resistance in MAC and *M. abscessus*,^37^ which is an important asset in multidrug treatment of NTM disease.^4,21^

With increased attention and publications, three roles for clofazimine were described in the 2020 and 2022 guidelines for NTM pulmonary disease: (i) clofazimine as a replacement for rifampicin or ethambutol in case of intolerability; (ii) adding clofazimine to regimens for severe or macrolide-resistant MAC-PD; and (iii) clofazimine as a preferred drug in the treatment of *M. abscessus* pulmonary disease. The guidelines suggest a dose of 100–200 mg per day, without justification.^4,5^

## Perspective

Why do we use a 100 mg once-daily dose of clofazimine in TB and NTM treatment? In the literature, there is no clearly stated rationale for this dose. Yet, six observations and programmatic preferences appear to have shaped this dosing strategy:

1. The 2 mg/kg dose was the lowest dose to achieve significant antimycobacterial effect in mice without clofazimine crystal formation in gut, liver, kidney spleen and lung tissue.^1^
2. Leprosy patients receiving higher mg/kg doses did not have better outcomes than those treated with lower mg/kg doses.^2,12^
3. Clofazimine was an expensive drug and higher doses would have been impossible in resource-poor settings where leprosy was endemic.^19^
4. Higher doses would lead to more adverse events, particularly skin hyperpigmentation.^16,19^
5. As a result of adverse events, cost and lack of evident dose–response relationship, investigators aimed to find the ‘minimal useful dose’.^14,19,26^
6. While the leprosy field preferred monthly supervised high doses, the TB field preferred steady once-daily dosing to be in line with other agents; the NTM field copied that approach. For clofazimine, such a dosing schedule was successfully used in the THELEP programme trials: 100 mg once daily.^18^

**Figure 1.**
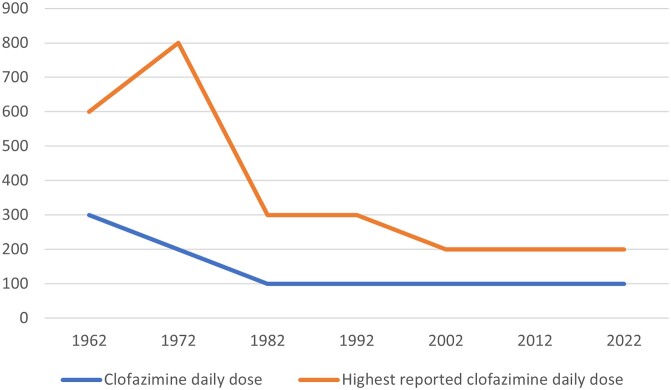
Recommended and highest reported daily clofazimine doses over time. Doses are given in mg/day. This figure appears in colour in the online version of *JAC* and in black and white in the print version of *JAC*.

The quest for the ‘minimal useful dose’^19,26^ was initiated immediately after the first evidence of clofazimine efficacy became available (Figure 1). While useful in the short term, i.e. to have the drug available for as many patients as possible, it may have been harmful in the long term as it may compromise clofazimine efficacy. The economical argument to stick with low doses is no longer relevant. It is important to critically assess the clofazimine dosing paradigms once more.

### Dose–response relationships for clofazimine in TB and NTM disease treatment

Dose–response relationships have been demonstrated in leprosy,^18^ but there are also studies reporting exposure–effect relationships in TB and NTM disease. In 105 patients with MDR-TB receiving clofazimine, an AUC/MIC ratio of >50 was associated with faster time to sputum culture conversion.^10^ A similar correlation between higher AUC/MIC ratios and 2 and 6 month culture conversion was recorded in 136 MDR-TB patients receiving clofazimine.^38^ A similar signal was observed in NTM disease. First, in mouse models of MAC and *M. abscessus* disease, inhaled clofazimine led to higher exposures and a stronger antimycobacterial effect in the lungs compared with conventional oral dosing.^39^ Second, three clinical studies in NTM pulmonary disease have observed relationships between exposure or susceptibility and treatment outcome. The first was a 38-patient retrospective analysis of mostly *M. abscessus* treatment, where outcomes of clofazimine-containing regimens were better if the MIC was ≤0.25 mg/L.^40^ The second study reported on 20 patients treated for MAC (*n* = 11) or *M. abscessus* (*n* = 9) with a 100 mg (*n* = 18) or 50 mg (*n* = 2) daily dose of clofazimine; again, the likelihood of culture conversion in patients with isolates with MIC ≤ 0.25 mg/L was much higher than in those with MIC > 0.5 mg/L (OR 39.3; *P* = 0.021).^41^ These signals are indirect, but again suggest that an exposure/MIC ratio is a driver of outcomes, and higher clofazimine exposures can thus improve outcomes. Recently, it was indeed observed in a clinical trial of 40 MAC-PD patients that peak serum clofazimine concentration (*C*_max_) and *C*_max_/MIC ratio predicted culture conversion.^42^

### Towards optimized dosing of clofazimine in TB and NTM disease

There is circumstantial evidence for a dose–response relationship for clofazimine. To better substantiate dosing in TB and NTM disease, pharmacodynamic models should confirm the pharmacodynamic index (now: AUC/MIC) and set AUC/MIC targets required for clofazimine to be active. Pharmacokinetic studies of different dosing regimens can then establish which doses are required for target attainment. A logical next step could be a maximum tolerated dose study—but these may in part have already been done. The early experiences in leprosy treatment have documented use of 300 mg daily doses for up to 12 months^2,12–14^ and 600 mg for 3 months;^14^ doses up to 800 mg for 3 to 6 months were applied in the clinical trial of clofazimine in management of Buruli ulcer disease (Figure 1).^41^ Although toxicity reporting was minimal in all these studies, this is a suggestion that such doses may be safe and tolerable.

Abdelwahab and colleagues^7^ have elegantly shown the benefit of a loading phase with 4 weeks of a ‘high’ 200 mg dose to achieve the steady state levels of clofazimine 3–6 weeks earlier, hoping that this would also lead to earlier efficacy of clofazimine and a stronger contribution to the efficacy of treatment regimens, in this case for TB. The pharmacokinetic importance of a loading phase is thus clear, but the exact dose remains to be established. The Abdelwahab study modelled loading phases followed by the 100 mg once-daily dose;^7^ the question is whether the loading phase should be followed up by a higher, perhaps 5 mg/kg (i.e. 200 or 300 mg in most adults) daily dose similar to the early leprosy trials.^2,12,13^ The ongoing move to shorter (2–4 months) TB treatment regimens^43^ further stresses that if clofazimine is to be used in future TB treatment regimens, its dosing needs to be increased significantly and it likely will need a loading phase, to achieve its maximal rather than minimal efficacy and in a time frame relevant to the new generation of short regimens. The safety, tolerability, pharmacokinetics and efficacy of such strategies should be subject to clinical trials.

One way of increasing exposure is to change the delivery to the site of infection; preclinical and clinical studies of an inhaled formulation of clofazimine are currently ongoing.^39^ It is important to realize that this may be more relevant to localized disease, i.e. NTM pulmonary disease, than to systemic diseases that manifest in the lung, such as TB.

### Toxicity—the great unknown?

Toxicity remains a concern, particularly for prolonged high doses of clofazimine. Clofazimine accumulates in body fat, skin, macrophages and the intestinal wall; the hyperpigmentation or skin discoloration seen with clofazimine may be worse, occur faster and take longer to resolve if exposures are increased. Reported patient perceptions of this skin colour change have always been mixed between ethnicities or skin types, ranging from ‘none of the patients was unhappy about this phenomenon; they were pleased to exchange pigmentation for chronic invalidism’^43^ to patients expressing fear of being treated with clofazimine and ‘turn an extremely unpleasant colour’.^13^ Patient perception of skin discoloration by clofazimine is an important area for qualitative studies. There are already attempts at creating novel r-iminophenazines that do not accumulate in the skin.^44^ QTc prolongation may also be a risk, particularly in patients with already prolonged QT intervals prior to clofazimine therapy.^15^ Perhaps more worrying and less studied is the clofazimine-induced enteropathy, which manifests with abdominal discomfort and diarrhoea, driven by crystal-storing histiocytosis;^45^ its late occurrence suggests that it, too, is an accumulation-driven phenomenon. If we are to reappraise high doses of clofazimine, we need to tread carefully as limited safety data are available.

In summary, the current 100 mg once-daily dose of clofazimine in TB and NTM treatment seems to be inherited from a deliberate attempt to find the minimum effective daily dose in leprosy treatment, later codified in the THELEP field trials. While this dose is safe, economical and practical, a higher dose with a loading phase may add relevant efficacy and treatment-shortening potential to both TB and NTM disease treatment. We need to revisit dose–response and maximum tolerated dose studies to get the best out of this drug, while continuing efforts to generate similar molecules that accumulate less in the skin and intestinal tissues and have simpler pharmacokinetic profiles that do not require the use of loading doses.
